# Re: Frisch & Simonson. Non-therapeutic male circumcision in infancy or childhood and risk of human immunodeficiency virus and other sexually transmitted infections: national cohort study in Denmark

**DOI:** 10.1007/s10654-022-00872-7

**Published:** 2022-06-20

**Authors:** Dan W. Meyrowitsch, Lena S. Andersen, Nicolai Lohse, My von Euler-Chelpin, Catherine Hankins

**Affiliations:** 1 Department of Public Health, University of Copenhagen, Copenhagen, Denmark; 2 Department of Neuroanaesthesia and Intensive Care, Copenhagen University Hospital Rigshospitalet, Copenhagen, Denmark; 3 Department of Epidemiology, Biostatistics, and Occupational Health, School of Population and Global Health, Faculty of Medicine and Health Science, McGill University, Montreal, Canada

The study recently published in the European Journal of Epidemiology by Frisch and Simonsen, which reports that non-therapeutic circumcision of boys in Denmark does not reduce risk of HIV infection and other sexually transmitted infections [1], does not consider sexual orientation in the interpretation of the observed findings. The study found that male circumcision was associated with higher rates of sexually transmitted infections overall, but this was driven by higher rates of anogenital warts and there was a very low occurrence of other STIs.

The authors do not consider or discuss that a man’s risk of anogenitally acquired sexually transmitted infections depends on his partner’s use of condoms and his partner’s circumcision status. It is not anatomically possible for a man’s circumcision status to influence his risk of STI/HIV acquisition if he is the receptive sexual partner in anal intercourse.

Low-risk genotypes of human papillomavirus (HPV) is the infectious agent responsible for causing anogenital warts. Only when the insertive partner is circumcised, conferring a lower risk of harbouring persistent HPV infection [2], will the risk of anogenital warts in the receptive partner be reduced if condoms are not used. Furthermore, a recent systematic review and meta-analysis in Lancet Global Health found that male circumcision has no effect on the risk of HIV acquisition among men who have sex with men (MSM) in high-income countries [3].

Since 2007, WHO and UNAIDS have recommended integrating male circumcision services into ongoing HIV prevention programs in 15 countries in sub-Saharan Africa. This recommendation is based on compelling evidence from rigorous randomised controlled trials conducted in South Africa, Kenya, and Uganda that demonstrated an approximately 60% reduction in heterosexually acquired HIV infection in men [4].

Since the data from Frisch and Simonsen were drawn from administrative databases, it was not possible to adjust for the role of sexual orientation and sexual-behavioural factors and this introduces a major limitation that undercuts the authors’ attempts to generalise their findings to voluntary medical male circumcision programs in Africa. In Denmark, cases of HIV among homosexual men during the period 1990–2004 accounted for 69.7% of all HIV infections among men [5], whereas in sub-Saharan Africa, HIV transmission is predominantly heterosexual.

The authors also focus on differentiating between male circumcision in infancy or childhood and adult male circumcision. However, there is no reason to believe that male circumcision in infancy or childhood will not result in equivalent HIV risk reduction for males, when they do become sexually active, as was seen in the randomised controlled trials among adult men. The biomedical explanatory models of friction-induced microtears allowing HIV access to susceptible target cells in the inner mucosal surface of the prepuce would account for the higher risk of HIV infection in uncircumcised males as compared to circumcised males, irrespective of whether their circumcision was carried out in childhood or later in life.

Attempting to generalize the findings of this observational study in Denmark to HIV prevention programs in sub-Saharan Africa, regardless of whether male circumcision is carried out in infancy, childhood or later in life, is potentially dangerous if it risks undermining effective HIV prevention programming for males.
